# Lifetime cardiovascular risk factors and maternal and offspring birth outcomes: Bogalusa Babies

**DOI:** 10.1371/journal.pone.0260703

**Published:** 2022-01-26

**Authors:** Emily W. Harville, Maeve E. Wallace, Hua He, Lydia A. Bazzano

**Affiliations:** 1 Department of Epidemiology, Tulane University School of Public Health and Tropical Medicine, New Orleans, LA, United States of America; 2 Department of Global Community Health and Behavior, Tulane University School of Public Health and Tropical Medicine, New Orleans, LA, United States of America; Univesity of Iowa, UNITED STATES

## Abstract

Both cardiovascular and reproductive complications may have origins in utero or in early life. Women in the Bogalusa Heart Study (n = 1401) had been linked to birth certificates for birthweight and gestational data, which were examined relative to childhood (ages 4–16) cardiometabolic indicators, indicated by mean levels overall and total risk factor burden as estimated by area under the curve (AUC) computed from longitudinal quadratic random-effects growth models. Women reported the birthweight and gestational age of each of their own pregnancies, and delivery medical records were linked to interview data where possible. Path analyses were conducted to examine the relationships among a woman’s own birth outcomes, childhood and preconception adult cardiovascular health, and birth outcomes. Mean blood pressure (systolic blood pressure (SBP) adjusted relative risk (aRR) per 1-SD increase, 1.27, 95% CI 1.04–1.57) and low-density lipoprotein (aRR 1.21, 95% CI 1.02–1.44) in childhood predicted preterm birth (PTB), while mean SBP (aRR 1.33, 95% CI 1.02–1.74) predicted term low birthweight. The AUC data suggested an association between blood pressure and PTB (aRR for SBP top 10%, 1.86, 95% CI 1.08–3.21). Pre-pregnancy total cholesterol was negatively associated with gestational age. In path analyses, positive associations were found for each step between own birthweight, childhood BMI, pre-pregnancy BMI, and child’s birthweight. Childhood levels of some, though not all, cardiovascular risk factors may predict adverse birth outcomes (preterm birth and reduced fetal growth).

## Introduction

Cardiovascular and reproductive health are closely linked. Developmental origins of health and disease theory links preconception exposures and birthweight to adult cardiovascular health. For instance, analyses in the Bogalusa Heart Study have indicated relationships between birthweight and adult blood pressure [1, 2], arterial stiffness [3], and metabolic syndrome [4]. Associations are also found between cardiovascular disease during pregnancy [5–8] or pre-existing, clinical cardiovascular conditions such as hypertension or diabetes and birth outcomes [9–11]. Women with chronic hypertension who become pregnant are at higher risk of preterm birth and small-for-gestational-age [12]. Some studies indicate that preconception cardiovascular health outside clinical disease also has an impact on pregnancy health; preconception risk factors linked to adverse pregnancy health include blood pressure [13], both low and high cholesterol [13, 14], and glucose [13].

Combining these lines of research, we hypothesized that a woman’s own birthweight and gestational age would also predict preconception and pregnancy health and that women with a higher burden of cardiovascular risk throughout childhood would have an increased risk of adverse birth outcomes (preterm birth and reduced fetal growth) (Conceptual model, S1 Fig). The Bogalusa Heart Study provides a unique opportunity to study birth outcomes within a life course and developmental origins framework. Few studies have examined the full preconception time frame, including childhood and adolescence. Baseline health or long-term damage may be better represented by multiple measures over time. Similarly, few studies have put preconception effects in context of a woman’s own birth outcomes.

## Methods

### Study population and data sources

The Bogalusa Heart Study (BHS) is a longitudinal study of cardiovascular risk founded by Dr. Gerald Berenson that began enrolling school children in the semi-rural community of Bogalusa, Louisiana in 1973 [15]. Approximately every 2 years thereafter, researchers repeated exams through 1994, enrolling new children each time and following up those previously enrolled. Exams included interview questionnaires, venipuncture after a 12-hour fast, right-arm blood pressure measures in triplicate, and replicate anthropometric measures. Further detail on BHS examinations is available elsewhere [15].

A follow-up study sought to determine the pregnancy histories and outcomes of women who had participated in the BHS (n = 5918). Three sources of information were potentially available: interview, medical records, and vital statistics. Potential participants were contacted via phone call, mailing or in person at the study field clinic. 1804 subjects were interviewed for 15–30 minutes regarding reproductive history, including the outcome of each pregnancy; the birthweight of all children; and whether each child was born early, late, or on time. For the purposes of this analysis, we limited the dataset to women who completed an interview in adulthood and had at least one BHS exam during their childhood (≤ age 16). The dataset was further restricted to women whose first birth was a singleton and occurred after age 16 to preserve temporality. The final sample included 1401 women with any childhood study visit, and 1052 with 2 or more visits during childhood (S1 Table; a flowchart is provided in S2 Fig and a schematic of the study design in S3 Fig).

Two additional sources of information were available. Participants were asked for permission to access medical records, and those consenting signed release forms for prenatal, labor and delivery records for each pregnancy. Almost all participants gave permission for forms to be accessed, although in many cases they had been destroyed as over 10 years old (most births occurred prior to widespread implementation of electronic medical records in Louisiana, and typical hospital policy was to retain paper records for no longer than 10 years). While 97% of women included in this analysis consented to release, medical records were obtained for only 10% of the sample (in most cases, because they had been destroyed as over 10 years old). Vital records data from Louisiana, Texas, and Mississippi were also linked to BHS data for 55% (n = 3260) of all women ever seen in the larger study, and an estimated 65% of all women expected to have given birth [16]. The strongest predictor of either linkage or interview was recent and/or frequent participation in the parent study [17]. Those who were interviewed had more study visits (median 5) than those who did not (median 2, p<0.01), and were more likely to have participated in the study as an adult. The groups that were interviewed were also more likely to have ever smoked. Parental education was more likely to be missing for those who were not located (this data was not collected at early visits); among those with data, those who were located were more likely to have higher parental education. Differences in BMI, cholesterol, and blood pressure were largely explained by the age distribution of participation in the groups, although mean childhood BMI was higher in those who only interviewed.

### Outcome

Adverse birth outcomes assessed included low gestational age (dichotomized as preterm birth, more than 3 weeks early or <37 weeks’ gestation) and birthweight. A measure of small-for-gestational-age (SGA) produced an unlikely proportion, most likely due to approximation of gestational age at term, so term low birthweight (TLBW; <2500 g) was examined as an indicator of reduced fetal growth.

Outcomes were taken from woman’s self-report. Where available, data on participant’s reproductive history as reported during the interview was compared with vital records and obstetric medical records in order to correct for misclassification of self-reports. Agreement between vital records and interview was good for participants who had data from both sources. After control for clustering within women, the mean discrepancy between the birth certificate and the woman’s report of birthweight was 2 g; for gestational age, the mean difference was 0.01 weeks [17]. The first singleton livebirth in the dataset was analyzed.

### Exposure

Exposures of interest included biological markers of cardiometabolic functioning in childhood (before age 16): body mass index (BMI, kg/m^2^), systolic and diastolic blood pressure (SBP and DBP, both mmHg), low-density lipoprotein cholesterol (LDL-C, mg/dL), high-density lipoprotein cholesterol (HDL-C, mg/dL), total cholesterol (TC, mg/dL), triglycerides, (mg/dL), insulin (uU/mL), and glucose (mg/dL). Serum cholesterol and triglycerides levels were assayed enzymatically on the Hitachi 902 Automatic Analyzer (Roche Diagnostics, Indianapolis, IN). Plasma glucose level was obtained as part of a multiple chemistry profile (SMA20) with the multichannel Olympus Au-5000 analyzer (Olympus, Lake Success, NY). A radioimmunoassay kit was used to measure plasma insulin (Phadebas insulin kit, Pharmacia Diagnostics, Piscataway, NJ).

### Covariates

Potentially confounding covariates known to be associated with both cardiometabolic health and pregnancy complications included maternal race (Black/White), age at delivery (continuous), educational attainment (less than high school, high school graduate, associate’s degree (AA) or some college, college graduate), marital status (married/not married) and smoking during pregnancy (yes/no). Models including non-BMI predictors were also adjusted for BMI at the visit prior to pregnancy. Related pregnancy complications assessed included preeclampsia, pregnancy-induced hypertension (PIH), and gestational diabetes (GDM). Although recall has been shown to be highly specific (>90%) for hypertensive disorders [18] and accurate for reports of gestational diabetes (GDM) (specificity = 98%, sensitivity = 92%) [19], we compared values to those reported in the medical record, where available, in order to ensure the most accurate case ascertainment. Following a hierarchy of validity, discrepant outcomes were resolved by classifying births based on non-missing data first from medical records, followed by maternal self-report where medical records were missing.

### Statistical analysis

Analysis proceeded in stages to test the conceptual model (S1 Fig). We considered the associations between maternal own birth outcomes and child-young adult cardiometabolic health to be sufficiently tested in BHS and other studies. [4, 20–24] To examine the relationship between preconception cardiometabolic health and pregnancy health, we estimated the effect of cardiovascular risk over time with different methods. First, we took the age-standardized value of each risk factor. If more than one measurement was available, the mean was used. This was then stratified at age 12.4, to approximate pre- and post-pubertal time periods (age at menarche was not available for enough of the sample to allow for individual classification of measures as pre- or post-pubertal; 12.4 was the median age at menarche among those with this datum.) All women with at least one childhood measure were included in this analysis (n = 1382 with complete data on covariates); a second analysis limited to those with visits both before and after age 12.4 (n = 728). Analysis of mean childhood measures before and after age 12.4 modeled the relative risk of dichotomous outcomes using log-Poisson models with a robust variance [25]. Adjusted models controlled for maternal age at delivery, race, education, marital status, lifetime ever smoking, and pre-pregnancy BMI.

Next, we computed the area under the curve (AUC) from longitudinal growth curve models, applying the methodology described in detail by Cook et al., in previous analysis of Bogalusa Heart Study data [26]. The AUC values represent standardized summary measures of childhood biomarkers estimated from repeated measures on the same individuals where measures did not take place at equally-spaced intervals [26]. We fit quadratic random-effects growth curves using PROC MIXED with a parameter for age centered around the approximate midpoint (age 10), as well as a quadratic age term to improve fit. The AUC was defined as the integral of the predicted biomarker curve from ages 4–16 divided by 10. AUC values for all 9 cardiometabolic indicators were computed for each individual based on a minimum of 2 measurements that occurred between age 4 and 16 and covariate data (n = 993).

We used the AUC for each biologic indicator to examine the association between childhood mean cardiometabolic profiles and risk of PTB and TLBW at first pregnancy. Chi-square and t-tests compared crude differences in women with an adverse birth outcome and those without across categorical and continuous covariates, respectively. Separate adjusted log-Poisson models estimated the relative risk associated with each cardiometabolic indicator controlling for adult social, biological, and behavioral factors, as indicated in our model [maternal age at delivery, race, education, marital status, pre-pregnancy BMI, and lifetime ever smoking (regularly and/or during pregnancy)]. All indicators were scaled to estimate relative risks associated with an interquartile range (IQR) increase in units. The group in the top 10% of the AUC was also compared to the others. Models were then examined with those with pregnancy complications removed, as well as adjusting for pregnancy complications, which addressed the possible associations with both pregnancy health and birth outcomes.

Finally, we considered the entire conceptual model. By conducting a path analysis, we are able to examine the complete life course of intercorrelations among own birth characteristics, childhood and preconception cardiovascular health, and offspring birth outcomes. Before path analysis, multivariable linear regression models were conducted to examine the associations at each step by regressing risk factors at current step on the risk factors at the previous step in the path. For each outcome under study, each childhood AUC measure was examined for association with maternal own birthweight outcomes and gestational age; childhood AUC measures were examined for associations with each cardiometabolic risk factor in pre-pregnancy; pre-pregnancy factors were examined for association with weight gain during pregnancy; and both pre-pregnancy and weight gain were examined for association with the outcome. Variable selections were conducted at each step, and factors associated with each other at each step at p<0.1 were further explored in a formal path analysis under structural equation modeling using either the likelihood method or Bayes method (when likelihood methods did not converge), with additional control for age, race, and maternal education. All variables meeting the criteria were included in the path analysis, and the results examined for which statistical associations remained. The path analysis was conducted using Mplus Version 7 [27].

This study was approved by the Institutional Review Board of Tulane University and all subjects provided written informed consent.

## Results

The study sample was 59% white, 41% black (Table 1). 17% reported smoking during pregnancy. Mean age at included pregnancy was 28 years. Women who were white, married, had more education, and did not smoke had a lower risk of adverse outcomes.

**Table 1:** Characteristics of the study population (n = 1401) all singleton first births occurring after age 16 among women with at least 1 BHS visits before age 16.

	Whole sample (n = 1401)	2 or more childhood visits (n = 1052)
	N(%)	N(%)
Race		
Non-white	575 (41.2)	425 (40.6)
White	820 (58.8)	621 (59.4)
Marital status		
Married	654 (46.8)	474 (45.2)
Non-married	743 (53.2)	575 (54.8)
Education		
Less than high school	129 (9.2)	89 (8.5)
high school graduate	406 (29.0)	298 (28.4)
Associate’s or some college	470 (33.6)	345 (32.8)
College or more	395 (28.2)	319 (30.4)
Smoking during pregnancy	233 (16.6)	176 (17.0)
Ever smoked	310 (22.1)	240 (22.8)
Preterm		123 (8.8)
Term low birthweight		68 (5.4)
	Mean (SD)	Mean (SD)
Age at pregnancy	23.2 (5.6)	23.0 (5.5)
Own birthweight	3219 (545)	3218 (559)
Own gestational age	39.6 (2.4)	39.6 (2.5)

*Numbers may not add to total due to missing data

The associations between childhood risk factors and adverse birth outcomes were mixed (Table 2). Associations were examined overall and for the time period before and after age 12.4. SBP, DBP, and LDL were associated with increased risk for PTB in the full sample. For TLBW, a positive association was seen with SBP and, in the group with measures from both time periods, was inverse association was seen with LDL. In most cases there were not differences in associations across the two time periods, with a few exceptions where measures taken prior to 12.4 years were stronger predictors of outcomes than measures taken later (for PTB, glucose in younger group adjusted relative risk [aRR] 1.55, 1.14–2.09, older aRR 1.08, 0.85–1.37; for tLBW, triglycerides in the younger group aRR 0.57, 0.34–0.95, older group 1.04, 0.74–1.46). Except for BMI, no overall AUC or top 10% of AUC for cardiovascular risk factors was associated with either birth outcome (Table 3). For BMI, being in the top 10% was associated with a reduced risk of PTB.

**Table 2:** Associations between adverse birth outcomes and cardiovascular risk factors among participants with early and late childhood measures, stratified by average age at puberty, the Bogalusa babies study.

	Preterm birth					Term low birthweight		
	RR^a^	95% CI	aRR^b^	95% CI	RR^a^	95% CI	aRR^b^	95% CI
BMI								
all participants (n = 1378)	1.08	0.91, 1.27	1.03	0.79, 1.34	1.03	0.82, 1.29	0.96	0.68, 1.34
participants with visits before and after age 12.4 (n = 726)	1.03	0.81, 1.32	1.06	0.71, 1.58	1.09	0.75, 1.60	0.95	0.46, 1.99
before age 12.4	1.00	0.77, 1.29	0.98	0.67, 1.42	1.07	0.72, 1.58	0.98	0.51, 1.88
equal to or after age 12.4	1.03	0.82, 1.28	1.08	0.72, 1.61	1.11	0.82, 1.51	0.95	0.50, 1.91
Systolic blood pressure								
all participants (n = 1378)	1.29	1.06, 1.58	1.27	1.04, 1.57	1.26	0.98, 1.62	1.33	1.02, 1.74
participants with visits before and after age 12.4 (n = 726)	1.33	1.00, 1.76	1.34	1.00, 1.80	1.12	0.72, 1.75	1.26	0.78, 2.04
before age 12.4	1.23	0.96, 1.57	1.26	0.97, 1.63	1.07	0.74, 1.55	1.17	0.77, 1.76
equal to or after age 12.4	1.24	0.99, 1.57	1.23	0.97, 1.56	1.09	0.76, 1.57	1.20	0.84, 1.73
Diastolic blood pressure								
all participants (n = 1378)	1.34	1.09, 1.64	1.32	1.08, 1.62	0.85	0.62, 1.16	0.96	0.69, 1.33
participants with visits before and after age 12.4 (n = 726)	1.50	1.13, 1.98	1.47	1.11, 1.96	0.64	0.42, 0.99	0.76	0.49, 1.16
before age 12.4	1.31	1.00, 1.71	1.31	1.01, 1.70	0.75	0.50, 1.11	0.79	0.51, 1.22
equal to or after age 12.4	1.32	1.08, 1.62	1.30	1.04, 1.61	0.80	0.56, 1.14	0.95	0.68, 1.32
LDL								
all participants (n = 1361)	1.23	1.02, 1.47	1.21	1.02, 1.44	0.97	0.75, 1.25	0.93	0.72, 1.19
participants with visits before and after age 12.4 (n = 702)	1.08	0.80, 1.48	1.08	0.81, 1.45	0.68	0.47, 0.99	0.65	0.45, 0.96
before age 12.4	1.07	0.80, 1.43	1.06	0.81, 1.38	0.71	0.50, 1.01	0.70	0.48, 1.03
equal to or after age 12.4	1.04	0.81, 1.36	1.06	0.82, 1.36	0.73	0.51, 1.04	0.69	0.50, 0.95
HDL								
all participants (n = 1362)	0.98	0.78, 1.23	0.98	0.77, 1.24	1.21	0.92, 1.59	1.16	0.87, 1.54
participants with visits before and after age 12.4 (n = 703)	1.00	0.76, 1.33	1.00	0.74, 1.34	1.48	0.97, 2.26	1.48	0.99, 2.20
before age 12.4	1.01	0.79, 1.30	1.03	0.79, 1.33	1.46	1.06, 2.02	1.49	1.07, 2.08
equal to or after age 12.4	0.97	0.80, 1.17	0.96	0.79, 1.16	1.21	0.84, 1.74	1.20	0.87, 1.65
Total cholesterol								
all participants (n = 1363)	1.23	1.03, 1.47	1.22	1.02, 1.44	1.04	0.80, 1.35	0.99	0.77, 1.27
participants with visits before and after age 12.4 (n = 704)	1.10	0.81, 1.48	1.09	0.82, 1.45	0.86	0.57, 1.30	0.87	0.59, 1.27
before age 12.4	1.10	0.84, 1.45	1.10	0.85, 1.42	0.92	0.64, 1.33	0.97	0.66, 1.34
equal to or after age 12.4	1.00	0.77, 1.31	1.01	0.77, 1.32	0.83	0.56, 1.22	0.81	0.57, 1.15
Triglycerides								
all participants (n = 1381)	1.06	0.90, 1.25	1.08	0.91, 1.29	0.76	0.54, 1.07	0.87	0.62, 1.22
participants with visits before and after age 12.4 (n = 704)	1.05	0.81, 1.38	1.05	0.80, 1.38	0.66	0.38, 1.14	0.74	0.42, 1.29
before age 12.4	1.15	0.94, 1.41	1.18	0.96, 1.46	0.50	0.29, 0.84	0.57	0.34, 0.95
equal to or after age 12.4	0.85	0.66, 1.09	0.83	0.65, 1.05	0.99	0.71, 1.38	1.04	0.74, 1.46
Insulin								
all participants (n = 856)	….^c^		1.18	0.99, 1.42	1.02	0.79, 1.32	0.91	0.67, 1.22
participants with visits before and after age 12.4 (n = 323)	1.20	0.80, 1.80	1.43	0.94, 2.16	1.14	0.66, 1.97	1.01	0.55, 1.86
before age 12.4	1.23	0.91, 1.65	1.40	1.00, 1.96	0.95	0.53, 1.73	0.93	0.57, 1.51
equal to or after age 12.4	0.99	0.77, 1.27	1.04	0.84, 1.29	1.13	0.87, 1.46	1.13	0.78, 1.62
Glucose								
all participants (n = 1251)	1.06	0.86, 1.30	1.06	0.86, 1.31	0.96	0.65, 1.41	1.06	0.73, 1.52
participants with visits before and after age 12.4 (n = 524)	1.49	1.05, 2.09	1.52	1.06, 2.18	1.00	0.47, 2.13	1.06	0.53, 2.12
before age 12.4	1.54	1.14, 2.07	1.55	1.14, 2.09	0.77	0.46, 1.31	0.82	0.47, 1.42
equal to or after age 12.4	1.09	0.87, 1.37	1.08	0.85, 1.37	1.16	0.65, 2.07	1.14	0.71, 1.83

^a^ RR associated with a 1-SD increase (age-standardized, mean value is more than one visit in time period).

^b^ adjusted for lifetime smoking, race, marital status, education, age, and pre-pregnancy BMI

^c^ failed to converge.

RR, relative risk; CI, confidence interval; BMI, body mass index; HDL, high-density lipoprotein; LDL, low-density lipoprotein.

**Table 3:** Adjusted RR and 95% CI for adverse birth outcomes associated with area under the curve measure of overall burden of childhood cardiovascular risk factors, adjusted for complications of pregnancy.

	preterm birth		term low birthweight	
	adjusted		adjusted	
	aRR	95% CI	aRR	95% CI
BMI				
IQR	0.70	0.48–1.01	0.98	0.57–1.68
top 10%	0.33	0.13–0.81	0.64	0.20–2.08
Systolic blood pressure				
IQR	1.14	0.87–1.50	1.14	0.77–1.68
top 10%	1.53	0.91–2.55	1.55	0.63–3.84
Diastolic blood pressure				
IQR	1.23	0.96–1.58	0.85	0.59–1.25
top 10%	1.33	0.78–2.27	0.24	0.03–1.75
LDL				
IQR	1.13	0.90–1.42	0.76	0.52–1.11
top 10%	1.11	0.61–2.03	0.44	0.11–1.78
HDL				
IQR	1.08	0.80–1.45	1.35	0.99–1.82
top 10%	1.56	0.83–2.96	1.49	0.68–3.27
total cholesterol				
IQR	1.17	0.93–1.46	0.96	0.66–1.39
top 10%	1.30	0.72–2.33	0.77	0.24–2.47
triglycerides				
IQR	0.91	0.75–1.10	0.87	0.64–1.19
top 10%	0.65	0.29–1.44	0.82	0.26–2.56
insulin				
IQR	1.03	0.82–1.27	0.94	0.78–1.14
top 10%	0.43	0.05–3.45	0.46	0.05–4.02
glucose				
IQR	1.02	0.66–1.57	1.14	0.64–2.04
top 10%	0.97	0.38–2.44	1.13	0.27–4.65

^a^ adjusted for lifetime smoking, race, marital status, education, age, and pre-pregnancy BMI

IQR, interquartile range; RR, relative risk; CI, confidence interval; BMI, body mass index; HDL, high-density lipoprotein; LDL, low-density lipoprotein; PTB, preterm birth; TLBW, term low birthweight.

**Fig 1:**
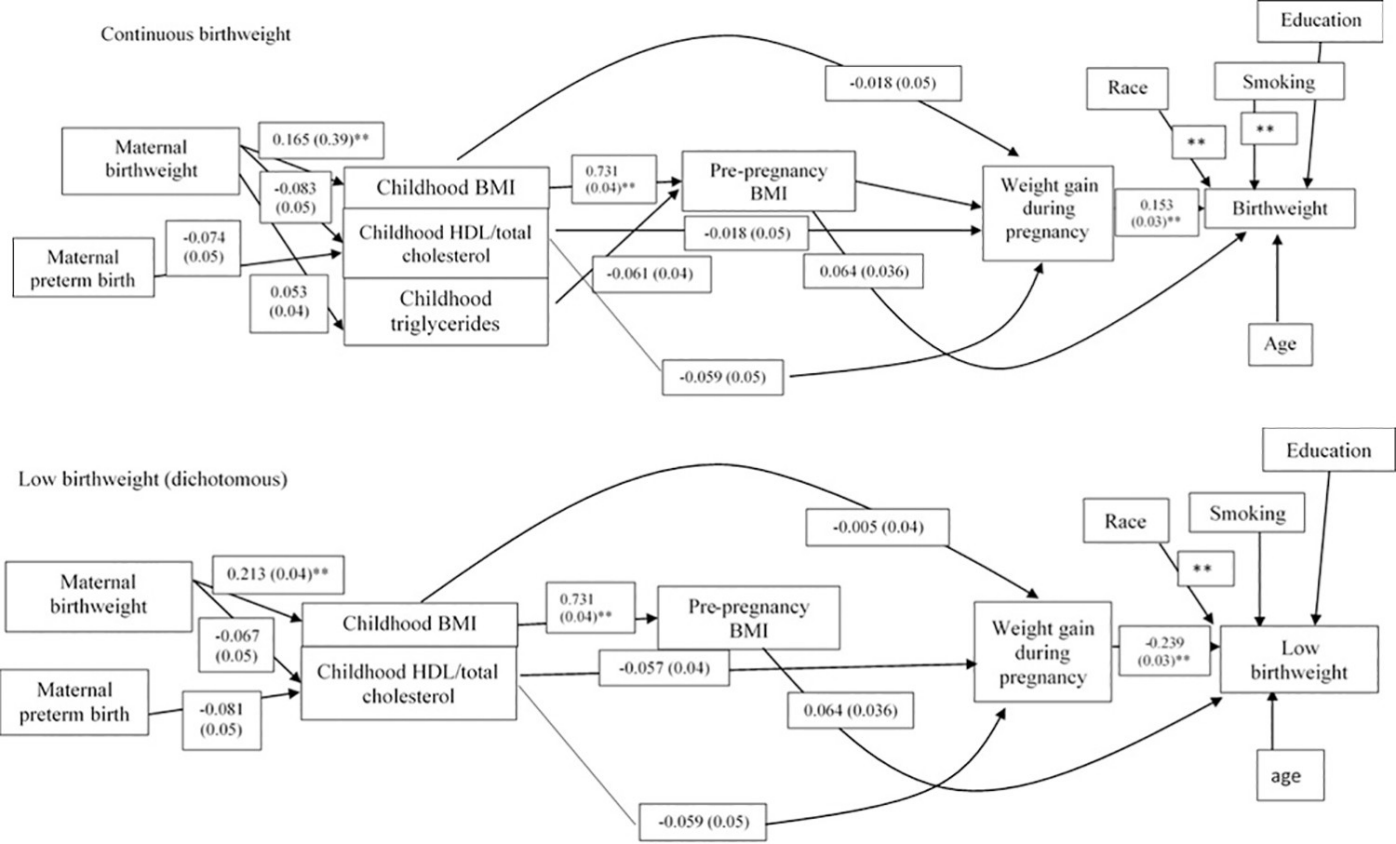
Path model for birthweight. Top panel, continuous birthweight; bottom panel, low birthweight. BMI, body mass index; HDL, high-density lipoprotein.

**Fig 2:**
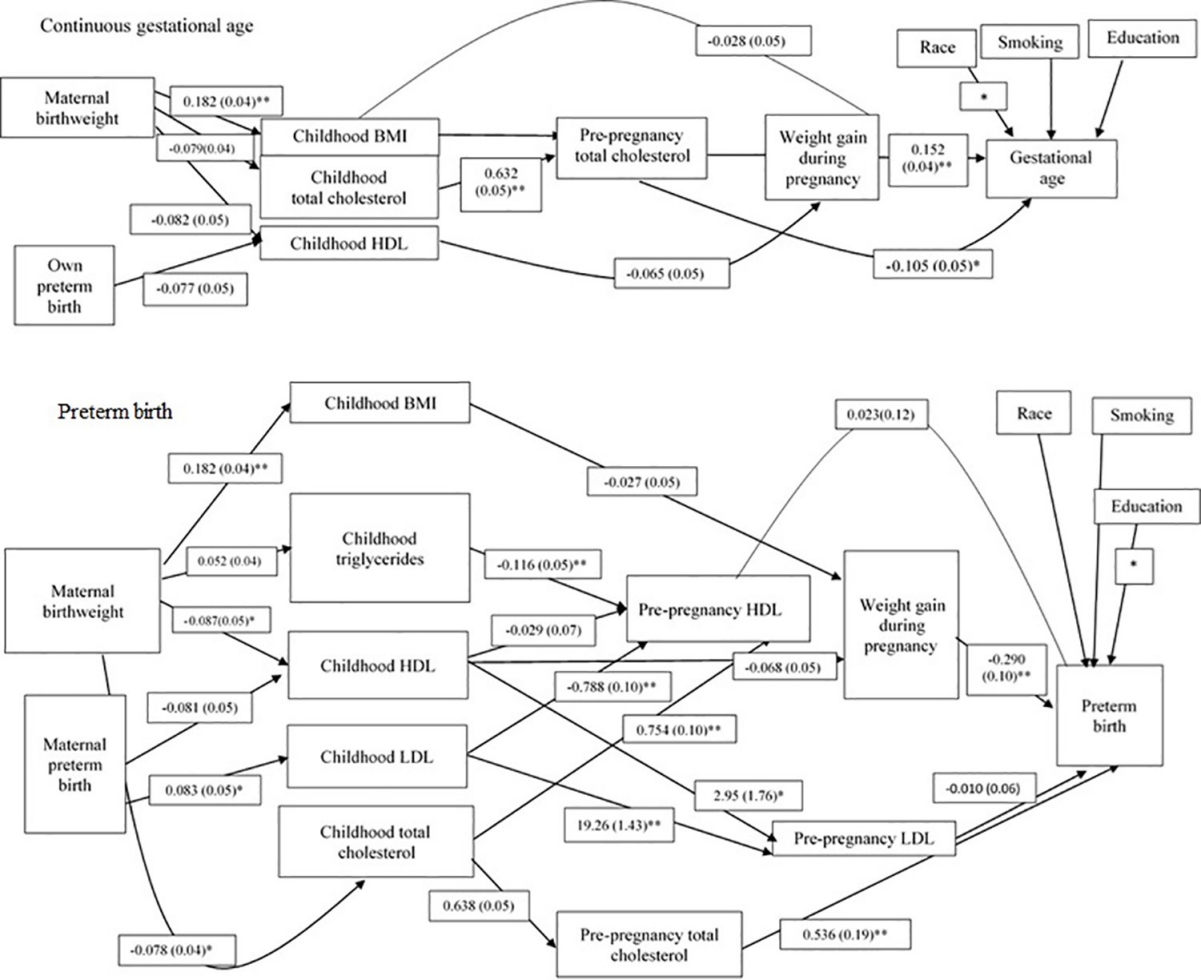
Path model for gestational age. Top panel, continuous gestational age; bottom panel, preterm birth. BMI, body mass index; HDL, high-density lipoprotein.

The path analysis is presented in Figs 1 and 2 and S2 and S3 Tables. The estimates and betas are provided along each path and in the tables, and p-values are additionally provided in the tables. The variables included in the models initially were those which were selected, in multivariable regression analysis, were associated with each other and p<0.1. In the full model for birthweight, the associations that remained were (moving from left to right in Fig 1, top panel) between maternal birthweight and childhood BMI, between childhood BMI and pre-pregnancy BMI, and between weight gain during pregnancy with birthweight. In the full model for low birthweight, the associations that remained were (moving from left to right in Fig 1, bottom panel) between maternal birthweight and childhood BMI, between childhood BMI and pre-pregnancy BMI, and between weight gain during pregnancy with birthweight. The associations that remained in the full model of gestational age (moving left to right in Fig 2, top panel) were: maternal birthweight was associated with childhood BMI, childhood cholesterol was associated with pre-pregnancy cholesterol, and total cholesterol was negatively associated with gestational age. Weight gain during pregnancy was also associated with gestational age. In the model of preterm birth (moving left to right in Fig 2, bottom panel), maternal birthweight was associated with childhood BMI and with childhood HDL, maternal preterm birth was associated with childhood LDL. Childhood triglycerides and LD were associated with pre-pregnancy HDL. Childhood HDL and LDL were associated with pre-pregnancy LDL childhood. Pre-pregnancy total cholesterol and weight gain during pregnancy were associated with preterm birth.

## Discussion

We examined the association of overall childhood and adolescent burden of cardiovascular risk with birth outcomes. Overall, higher childhood blood pressure was associated with a higher risk of PTB. Higher LDL cholesterol was associated with higher risk of PTB and lower risk of tLBW. Other risk factors had no or inconclusive associations.

We therefore found a degree of support, but not full confirmation, for our conceptual model. Central to this analysis was examining the associations between preconception (childhood, adolescence, and early-adult) cardiometabolic health and birth outcomes. While other studies have similarly found higher preconception blood pressure was associated with lower birthweight and higher preterm birth [13, 28], previous research on lipids is more ambiguous. Our previous analysis of the association between blood pressure, lipids, and birth outcomes in the Cardiovascular Risk in Young Finns Study indicated effect sizes between 1.2 and 1.4 [29]. The results of these studies are consistent with this, although precision of estimates varies. In some analyses, we also found higher triglycerides associated with lower risk of tLBW and higher HDL was associated with higher risk of tLBW. In the complete sample, higher total cholesterol was associated with PTB. Romundstad et al. found higher preconception levels of cholesterol and triglycerides and lower HDL were associated with higher birthweight [13], while Catov et al. found that both low and high total cholesterol were associated with increased preterm birth risk [14], but not triglycerides, LDL, and HDL. Therefore, our results are broadly consistent with these studies. Similarly, studies indicate that preconception glucose is generally associated with higher birthweight [13, 30]; however, in our study, the only relationship we found was between early-life glucose and higher risk of PTB. However, our sample size for insulin and glucose is substantially smaller than for blood pressure and lipids.

When the life course pathway and conceptual model was considered as a whole (birth→childhood→pre-pregnancy→pregnancy→birth outcomes), associations could be seen for a path between birthweight, childhood BMI, adult BMI, weight gain, and child’s birthweight. Other pathways that started with own birth outcomes and led through childhood and pre-pregnancy cardiometabolic risk factors to offspring birth outcomes were minimal. Our conceptual model was therefore, again, supported to a degree but not fully. We are unaware of other studies that have been able to address a complete life course pathway. However, many developmental origins studies have found low and high birthweight or prematurity to be associated with adult hypertension, triglycerides, or diabetes [31–33]. Some studies have addressed the path starting from birth outcomes through childhood to early-life blood pressure [34].

Lifetime cardiovascular risk could have effects on pregnancy in several ways. One, it could simply represent cardiovascular health during the pregnancy. Women with hypertension, dyslipidemia, and diabetes have a higher risk for poor pregnancy outcomes [35, 36]. Subclinical cardiovascular health might also be important; for instance, poorer cardiovascular health may incorporate endothelial dysfunction, which causes uteroplacental vasculopathy and issues of placental perfusion [37]. Second, pre-pregnancy cardiovascular risk could represent cardiovascular health during very early pregnancy, perhaps affecting placentation or other aspects of early pregnancy health. Changes in cardiovascular function occur very early in pregnancy [38], and are not necessarily captured by pregnancy-specific studies, but might still be important to pregnancy outcomes. Third, there may be underlying phenotypes or mechanisms that affect both cardiovascular and pregnancy health, including proatherogenic states, lipid metabolism, nutritional status, or susceptibility to infection [37]. Finally, pre-pregnancy cardiovascular health could have an independent effect on birth outcomes, perhaps by causing alterations in endocrine or inflammatory biology, or epigenetic changes, that would affect pregnancy outcomes.

This study examined relationships between cardiovascular risk and birth outcomes within a single generation, but the associations may go beyond this. Maternal cardiovascular health contributes to her child’s pre and postnatal environment, which subsequently affects the preconception and prenatal environment for the subsequent generation, and even beyond. Population-based three-generational effects on birth outcomes have been demonstrated. Grandparents’ (all four) higher incidence of cardiovascular mortality was associated with lower grandchild birthweight in a very large study in Norway [39]; U-shaped associations with maternal grandmother’s diabetes mortality risk were seen for grandchild birthweight, while for all other grandparents, higher diabetes mortality was associated with lower birthweight [39]. Several vertebrate studies have also found transgenerational relationships among birth outcomes, adiposity, and glucose/insulin metabolism, including a low protein diet in generation 1 in rats being associated with increased glycemia in generation 2 and high birthweight in generation 3 [40], and increased Generation 3 birthweight among mice whose grandparents had gestational diabetes [41].

Strengths of the study include the standardized protocol for measuring cardiovascular risk factors, a prospective study design, and a community-based, unselected (not referred from clinic or high-risk) population. Most previous studies that have examined preconception health have been limited to Scandinavian populations [13, 29, 30] or adults [14], usually considering only a single time point. The cohort is biracial and includes participants from both races with a range of educational attainment. Limitations include the amount of variation in number and timing of visits. Child development is not uniform, and measures taken four years apart could have very different implications depending on whether they are taken at age 4 and 8 or 10 and 14. We attempted to deal with this limitation analytically, but it cannot be eliminated. The lack of information on cardiovascular health during the pregnancy limits the conclusions we can draw about the strength of an effect independent of pregnancy health. Also, outcomes were self-reported; birthweight and gestational age have generally been indicated to be well-reported by mothers [42], but, particularly for gestational age (which women often remember in terms of weeks instead of days), it could incorporate error. Statistically, overall rates of preterm birth and low birthweight in this sample are lower than would be expected, which also limits our ability to detect smaller associations, and false positives are possible, given the relatively large number of tests. Birthweight and gestational age are very approximate measures of morbidity, and affected by a range of factors both prenatal and perinatal, including clinical interventions. In addition, limited concurrent socioeconomic measures, such as income, occupation, and wealth, were available to incorporate into the analysis. Cardiometabolic health could be a mechanism by which social factors translate to adverse birth outcomes, in which case control for these factors would not be appropriate.

The implications of study results for public health should be considered. Interventions—improving cardiovascular health for both children and young women—would be likely be similar regardless of mechanism by which pre-pregnancy cardiovascular health has its effect. Determining the precise causal mechanism of these associations and thus the appropriate timing of interventions is an important area for future research. However, given the large proportion of unplanned pregnancies [43], general improvement in cardiovascular health, rather than targeted preconception care, may be most effective in impacting health for both adults and their infants.

## Supporting information

S1 Table Number of visits and age at each visit, study sample (n = 1401). (DOCX)

S2 Table Path analysis of birthweight. (DOCX)

S3 Table Path analysis of gestational age. (DOCX)

S1 Fig Conceptual model. (DOCX)

S2 Fig Study population. (DOCX)

S3 Fig Study design. (DOCX)

S1 File (DOCX)

## Abbreviations

BMI: body mass index
LDL: low-density lipoprotein
HDL: high-density lipoprotein
LBW: low birthweight
TLBW: term low birthweight
PTB: preterm birth
SGA: small-for-gestational-age
AUC: area under the curve
IUGR: intrauterine growth restriction
BHS: Bogalusa Heart Study
SBP: systolic blood pressure
DBP: diastolic blood pressure
IQR: interquartile range
RR: relative risk
aRR: adjusted relative risk
CI: confidence interval
