# Supplementary material for: Lifetime cardiovascular risk factors and maternal and offspring birth outcomes: Bogalusa Babies
Source: PLoS One. 2022 Jan 26;17(1):e0260703. doi: 10.1371/journal.pone.0260703 (PMC8791492; doi:10.1371/journal.pone.0260703)
Supplement: S1 Table — (DOCX) [file pone.0260703.s001.docx]

| S1 Table. Number of visits and age at each visit, study sample (n=1401) | | | | | | | | | | | | | | | | | | | | | |  |
| --- | --- | --- | --- | --- | --- | --- | --- | --- | --- | --- | --- | --- | --- | --- | --- | --- | --- | --- | --- | --- | --- | --- |
|  |  |  |  |  | first visit | | | | |  | median visit | | | | |  | oldest visit (age 16 or under) | | | | | |
| Number of visits |  | N | % |  | mean age | median age | p25 | p75 | range |  | mean age | median age | p25 | p75 | range |  | mean age | median age | p25 | p75 | range | |
| 1 |  | 349 | 24.9 |  | 11.1 | 11.7 | 8.3 | 13.6 | 4.8-15.9 |  |  |  |  |  |  |  |  |  |  |  |  | |
| 2 |  | 344 | 24.6 |  | 9.9 | 9.8 | 7.1 | 12.5 | 4.6-15.0 |  |  |  |  |  |  |  | 13.5 | 14 | 11.9 | 15.4 | 6.7-16.0 | |
| 3 |  | 330 | 23.6 |  | 7.5 | 7.1 | 6 | 8.8 | 4.9-14.8 |  | 10.5 | 10.3 | 9.4 | 11.9 | 6.2-14.8 |  | 13.7 | 13.8 | 12.6 | 14.9 | 7.4-16.0 | |
| 4 |  | 274 | 19.6 |  | 7.5 | 6.9 | 5.9 | 8.1 | 3.8-12.0 |  | 10.9 | 10.8 | 9.8 | 11.6 | 7.1-14.5 |  | 14.6 | 14.8 | 14.2 | 15.3 | 8.7-16.0 | |
| 5+ |  | 104 | 7.4 |  | 7.6 | 8.1 | 5.7 | 8.7 | 4.5-12.0 |  | 10 | 10.3 | 8.3 | 10.8 | 7.2-13.9 |  | 14.1 | 13.9 | 13.5 | 10.8 | 7.2-13.9 | |
