# Supplementary material for: Lifetime cardiovascular risk factors and maternal and offspring birth outcomes: Bogalusa Babies
Source: PLoS One. 2022 Jan 26;17(1):e0260703. doi: 10.1371/journal.pone.0260703 (PMC8791492; doi:10.1371/journal.pone.0260703)
Supplement: S2 Table — (DOCX) [file pone.0260703.s002.docx]

S2 Table. Path analysis of birthweight

| Path – birthweight | estimate (SE) | p-values |
| --- | --- | --- |
|  |  |  |
| birthweight-child BMI | 0.165 (0.39) | <0.01 |
| child HDL-weight gain | -0.059 (0.05) | 0.21 |
| own birthweight-child HDL | -0.083(0.05) | 0.07 |
| own preterm birth – child HDL | -0.074 (0.05) | 0.15 |
| birthweight – child triglycerides | 0.053(0.04) | 0.22 |
| child BMI – weight gain in pregnancy | -0.018(0.05) | 0.71 |
| child HDL – weight gain in pregnancy | -0.059(0.05) | 0.21 |
| child BMI- pre-pregnancy BMI | 0.731(0.04) | <0.01 |
| child triglycerides – pre-pregnancy BMI | -0.061 (0.04) | 0.12 |
| pre-pregnancy BMI-birthweight | 0.064 (0.036) | 0.07 |
| weight gain-birthweight | 0.153(0.03) | <0.01 |
| age-birthweight | -0.444(0.35) | 0.20 |
| Education-birthweight | 0.017 (0.03) | 0.58 |
| Race-birthweight | -0.324 (0.06) | <0.01 |
| Smoking-birthweight | -0.232(0.07) | <0.01 |
| **low birthweight** |  |  |
| birthweight-child BMI | 0.213 (0.04) | <0.01 |
| child HDL-weight gain | -0.059 (0.05) | 0.21 |
| own birthweight-child HDL | -0.067(0.05) | 0.16 |
| own preterm birth – child HDL | -0.081(0.05) | 0.09 |
| child BMI – weight gain in pregnancy | -0.005(0.04) | 0.90 |
| child HDL – weight gain in pregnancy | -0.057(0.04) | 0.20 |
| weight gain-LBW | -0.239(0.06) | <0.01 |
| Education-LBW | 0.015(0.09) | 0.87 |
| Race-LBW | 0.506(0.17) | <0.01 |
| Smoking-LBW | 0.227(0.18) | 0.20 |

BMI, body mass index; HDL, high-density lipoprotein; LBW, low birthweight
