# Supplementary material for: Lifetime cardiovascular risk factors and maternal and offspring birth outcomes: Bogalusa Babies
Source: PLoS One. 2022 Jan 26;17(1):e0260703. doi: 10.1371/journal.pone.0260703 (PMC8791492; doi:10.1371/journal.pone.0260703)
Supplement: S3 Table — (DOCX) [file pone.0260703.s003.docx]

S3 Table. Path analysis of gestational age

| Path | estimate (SE) | p-values |
| --- | --- | --- |
| **gestational age** |  |  |
| own birthweight-child cholesterol | -0.079 (0.04) | 0.06 |
| child BMI-own birthweight | 0.182 (0.04) | <0.01 |
| own birthweight-child HDL | -0.082(0.05) | 0.08 |
| own preterm birth – child HDL | -0.077 (0.05) | 0.13 |
| child BMI – weight gain in pregnancy | -0.028(0.05) | 0.55 |
| child HDL – weight gain in pregnancy | -0.065(0.05) | 0.17 |
| pre-pregnancy cholesterol-child cholesterol | 0.632(0.05) | <0.01 |
| weight gain-gestational age | 0.152(0.04) | <0.01 |
| cholesterol-gestational age | -0.105(0.05) | 0.03 |
| Education-gestational age | -0.056(0.04) | 0.21 |
| Race-gestational age | -0.181(0.09) | 0.04 |
| Smoking-gestational age | -0.109(0.11) | 0.30 |
|  |  |  |
| **preterm birth** |  |  |
| own birthweight-child total cholesterol | -0.078 (0.04) | 0.06 |
| own birthweight – child triglycerides | 0.052 (0.04) | 0.24 |
| own birthweight-child HDL | -0.087 (0.05) | 0.06 |
| own preterm birth – child HDL | -0.081 (0.05) | 0.12 |
| own preterm birth – child LDL | 0.083 (0.05) | 0.06 |
| own birthweight – child BMI | 0.182 (0.04) | <0.01 |
| child HDL – pre-pregnancy HDL | -0.029 (0.07) | 0.68 |
| child LDL – pre-pregnancy HDL | -0.788 (0.10) | <0.01 |
| child cholesterol – pre-pregnancy HDL | 0.754 (0.10) | <0.01 |
| child triglycerides – pre-pregnancy HDL | -0.116 (0.05) | 0.02 |
| child cholesterol – pre-pregnancy total cholesterol | 0.638 (0.05) | <0.01 |
| child HDL – pre-pregnancy LDL | 2.95 (1.76) | 0.10 |
| child LDL – pre-pregnancy LDL | 19.26 (1.43) | <0.01 |
| child BMI – weight gain during pregnancy | -0.027(0.05) | 0.56 |
| child HDL – weight gain during pregnancy | -0.068 (0.05) | 0.14 |
| weight gain during pregnancy – PTB | -0.290(0.10) | <0.01 |
| pre-pregnancy LDL – PTB | -0.010 (0.01) | 0.12 |
| pre-pregnancy cholesterol – PTB | 0.536 (0.19) | <0.01 |
| pre-pregnancy HDL – PTB | 0.023 (0.12) | 0.86 |
| Education – PTB | 0.163 (0.09) | 0.08 |
| Race – PTB | -0.024 (0.18) | 0.90 |
| Smoking – PTB | -0.236 (0.25) | 0.34 |

PTB, preterm birth; BMI, body mass index; HDL, high-density lipoprotein; LDL, low-density lipoprotein
