# Supplementary figures and images for: Lifetime cardiovascular risk factors and maternal and offspring birth outcomes: Bogalusa Babies
Source: PLoS One. 2022 Jan 26;17(1):e0260703. doi: 10.1371/journal.pone.0260703 (PMC8791492; doi:10.1371/journal.pone.0260703)

S1 Fig. Conceptual model


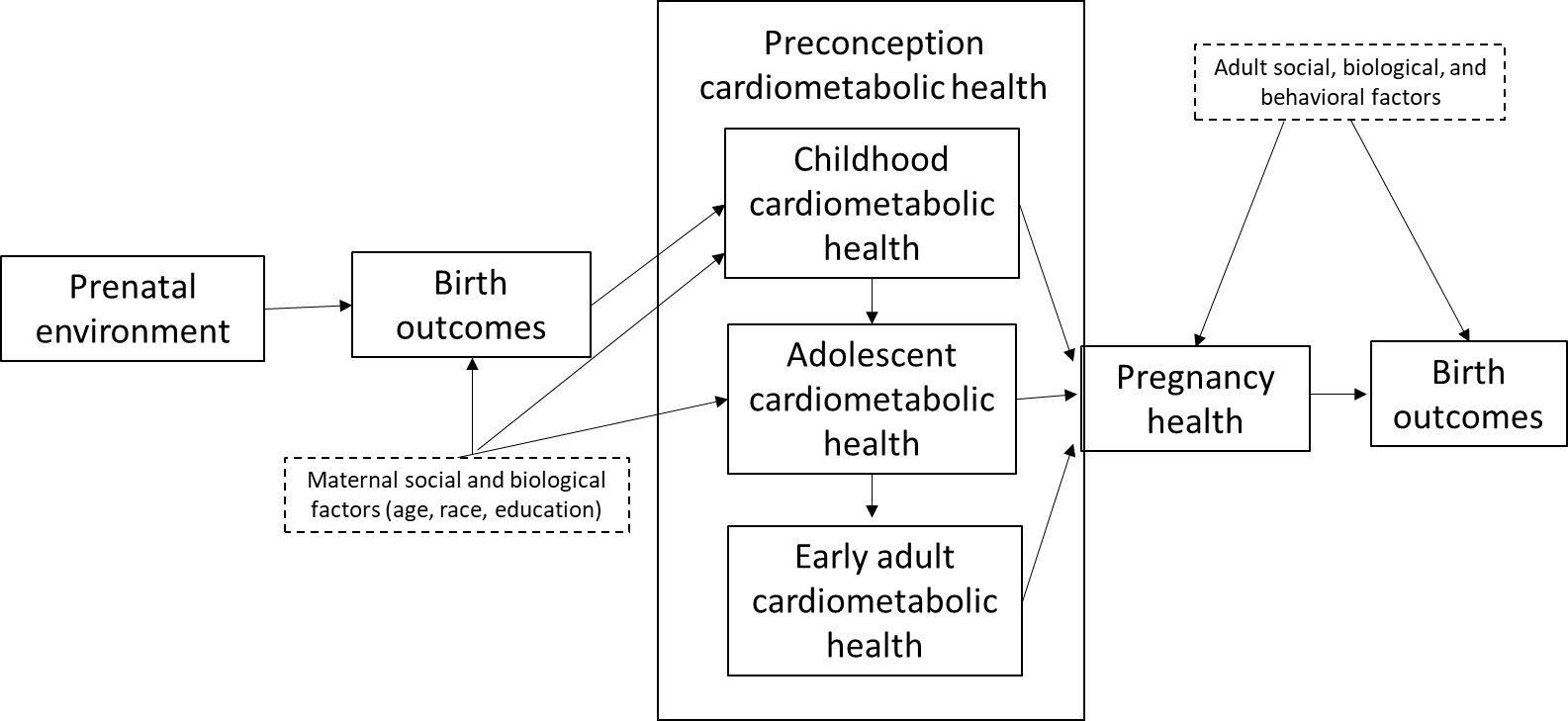

Supplement: S1 Fig — (DOCX) [file pone.0260703.s004.docx]

S3 Fig. Study design


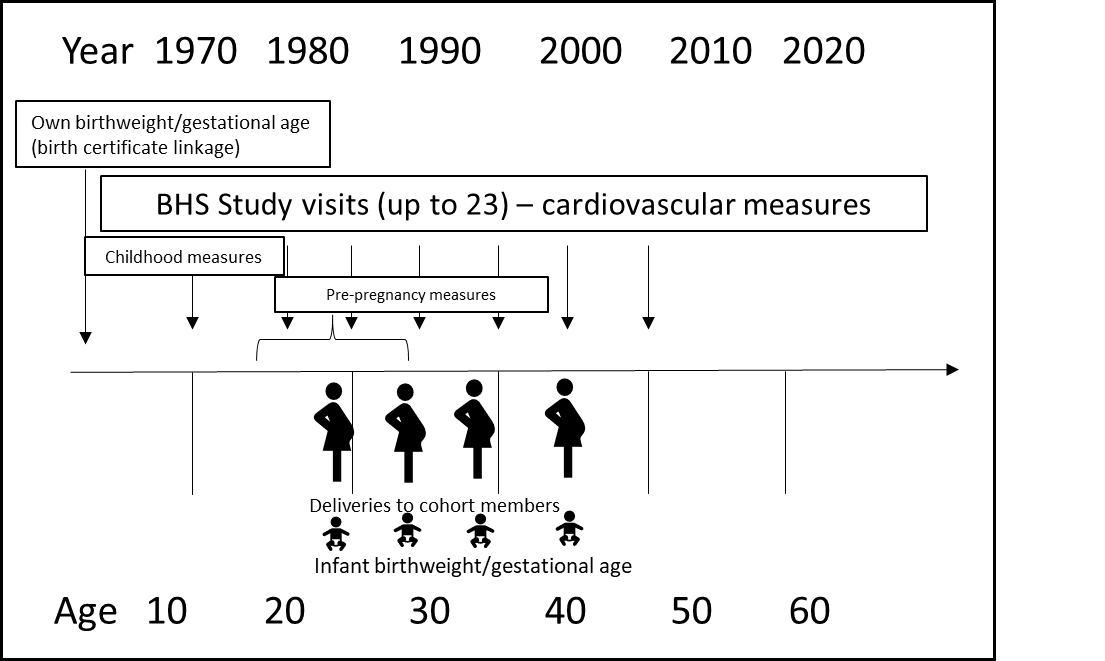

Supplement: S3 Fig — (DOCX) [file pone.0260703.s006.docx]
