# Supplementary material for: Lifetime cardiovascular risk factors and maternal and offspring birth outcomes: Bogalusa Babies
Source: PLoS One. 2022 Jan 26;17(1):e0260703. doi: 10.1371/journal.pone.0260703 (PMC8791492; doi:10.1371/journal.pone.0260703)
Supplement: S2 Fig — (DOCX) [file pone.0260703.s005.docx]

S2 Fig. Study population

728 with visits before and after age 12.4

5918 women/girls who ever participated in BHS

1804 participated in Bogalusa Babies substudy

1401 women with singleton first birth after age 16 and study visit before age 16

1052 with 2 or more childhood visits

1545 parous women

1525 with singleton first births and birthweight or gestational age data

1382 with complete data on covariates

993 with AUC calculation and data on covariates

Analysis of pre-pregnancy risk factors and birth outcomes (table 2)

Analysis of both pre- and post-pubertal risk factors and birth outcomes (table 2)

Analysis of entire childhood as AUC of risk factors (Table 3)

Path analysis (Figures 1 and 2, Tables S2 and S3)

630 with at least one own birth outcome data, childhood data, preconception data, and offspring birth outcomes data
