# Supplementary material for: Lifetime cardiovascular risk factors and maternal and offspring birth outcomes: Bogalusa Babies
Source: PLoS One. 2022 Jan 26;17(1):e0260703. doi: 10.1371/journal.pone.0260703 (PMC8791492; doi:10.1371/journal.pone.0260703)
Supplement: S1 File — (DOCX) [file pone.0260703.s007.docx]

**Pregnancy history questions**

1. Have you ever been pregnant? Please say yes even if the pregnancy was not carried to term.

Yes

No → Go to question 62

Not sure🡪 Why?

Think might be currently pregnant, but not sure 🡪 reschedule interview

Think might have had miscarriage 🡪 question 2 and follow miscarriage prompts

Other vagueness 🡪 skip pregnancy questions

I’d like to ask you a few questions about when you were pregnant.

1. When was the first time you got pregnant?

Age _______ or year ______

1. Did this pregnancy end in a livebirth, a stillbirth, a miscarriage, or something else, or are you still pregnant?

Still pregnant 1 🡪 question 4

Livebirth 2 🡪 question 6

Stillbirth 3 🡪 question 6

Miscarriage 4 🡪 question 52

Abortion 5 🡪 question 59

Other (molar/ectopic) 6 🡪 question 52

1. When was your last menstrual period? __/__/____
2. When is your due date? (if currently pregnant) __/__/__
3. Do you/Did you have a single baby, twins, or triplets?

Singleton

Twins🡪 repeat questions 8-9 for each baby

Triplets 🡪 repeat questions 8-9 for each baby

1. When was your baby born?__/__/____ (confirm with answer to #2)

8. Was it a boy or a girl?

Boy

Girl

What is his/her name?____________

1. What was his/her weight at birth? ___ lb. ___ oz. or ____g
2. When *name* was born, was s/he born before his/her due date, after his/her due date, or on the due date?

Before

After

On time🡪question 12

1. How many days or weeks early or late was s/he? ____ days ___ weeks
2. Did you ever breastfeed or pump breast milk to feed *name*, even for a short period of time?

Yes

No

Not Sure

1. How old was *name* when s/he *completely* stopped breastfeeding or being fed breast milk?
2. How old was *name* when s/he was first fed formula?

15. This next question is about the first thing that *name* was given other than breast milk or formula. Please include juice, cow’s milk, sugar water, baby food, or anything else that *name* may have been given, even water. How old was *name* when s/he was first fed anything other than breat milk or formula?

**Complications and health behaviors module**

I’m going to ask you a few more questions about your pregnancy with *name* [or ending *year*.] Please tell me just about that pregnancy – I will ask you about the other times you have been pregnant later.

16. During this pregnancy, did the doctor ever tell you that you had diabetes (gestational diabetes)?

Yes

No

Not sure

17. During this pregnancy, did the doctor ever tell you that you had hypertension/high blood pressure (gestational hypertension)?

Yes

No

Not sure

18. During this pregnancy, did the doctor ever tell you that you had pre-eclampsia, eclampsia, or toxemia?

Yes

No

Not sure

19. During this pregnancy, did the doctor ever tell you that you had protein in your urine?

Yes

No

Not sure

20. Did you smoke during the first three months of this pregnancy?

Yes

No

Not sure

21. Did you smoke during the second three months or end of this pregnancy?

Yes

No

Not sure

Not yet in the second trimester

22. How much weight have you gained/did you gain during this pregnancy?

None or lost weight

<10 lbs.

10-20 lbs.

20-30 lbs.

30-40 lbs.

40-50 lbs.

50-60 lbs.

60-70 lbs.

70-80 lbs.

>80 lbs.

Again, I’m asking you just about your pregnancy with *name* [or ending *year*.]

23. Were you or your partner doing anything to prevent pregnancy the month you got pregnant, that is, were you using any birth control methods?

Yes🡪 question 26

No

Not sure

24. How many months did you have unprotected sex before you got pregnant? If you got pregnant the first month, please say 1 month.

____

25. Did you take any fertility drugs or receive any medical procedures from a doctor, nurse, or other health care worker to help you get pregnant? (This may include infertility treatments such as fertility-enhancing drugs or assisted reproductive technology.)

Yes 🡪specify________________

No

26. Were you married when the baby was born?

Yes🡪question 28

No

Not sure

27. Were you living with a partner when the baby was born?

Yes

No

Not sure

**Providers module**

28. Did you give birth in a hospital, at home, or somewhere else?

Hospital

Home

Somewhere else

29. What was the name of the hospital? ______________

If not one of expected list 🡪 do you remember the address or where it is located?_____________________

30. Did you deliver with a doctor, a midwife, a nurse practitioner, or someone else?

Doctor

Midwife

Nurse practitioner

Someone else 🡪 specify___________

31. Did you go into labor spontaneously, or did you have your labor induced? In other words, did your labor start by itself, or did the doctor give you some medicine to get it started?

__ Spontaneous

__Induced

__Other 🡪 specify

31. Did you give birth vaginally, or by Caesarean section? [*If necessary*, By cutting the baby out, an operation through the stomach]

Vaginal

C-section 🡪31a

Something else🡪 specify______________________________

Not sure

31a. Did you plan to have a Caesarean section?

Yes

No

Unsure

31 b. Did you go into labor – either spontaneously or through an induction - before you had the c-section?

Yes

No

Unsure

32. What was the name of your *doctor/midwife/etc*.?_________________

We would like to know more about your health during the pregnancy and delivery and your baby’s health after delivery. For that reason, we are interested in looking at your medical records. You would need to give us permission to do this. We are only interested in records of your delivery and prenatal care and your baby’s care in the nursery.

If you give us permission to look at your records, we will mail a form to you for you to complete, sign, and return to us. This form gives your doctor permission to release your pregnancy and delivery records and your baby’s nursery records to us. Your doctor will not release your records to us without this permission form.

Willing or considering🡪 question 33

Not willing 🡪question 45

33. [When you were at the hospital for your delivery], did you see any other doctors beside Dr. *name*?

Yes-🡪What was his/her name?______________ _______________ (repeat questions 33 through 37 for each provider)

No

Not sure

34. Some *doctors/midwives/etc*. work with other doctors or at a central clinic. For instance, you might have seen Dr. Striplin at Total Women Care, or you might have seen a midwife who worked with Dr. Labadie’s practice. Did Dr. *name* work with a practice or group of doctors?

Yes 🡪 What was the name of the *practice/clinic*? ____________

No, Dr. *name* worked alone

Not sure

35. Where was their office located?_________________(as specific as possible)

36. Do you happen to know the phone number for the *clinic*?_______________

37. How about an email or website address?____________________________

38. Did you get prenatal care for this pregnancy (ending in *year*/with *name of child*)?

Yes

No 🡪 question 45

Not sure

39. Did you see Dr. *name* for prenatal care as well as for your delivery?

Yes🡪 question 45

No

Not sure

40. What was the name of the *doctor/midwife/nurse-practitioner* that you saw for prenatal care?___________________________

42. Do you happen to know the phone number for the *clinic*?________________

43. How about an email or website address?______________________________

44. Did you see any other *doctor/midwife/nurse-practitioners* for prenatal care?

Yes🡪 question 40; repeat 40-43 for all providers

No

Not sure

45. Around the time you were pregnant, did you see a doctor for reasons other than the pregnancy? (If you saw more than one doctor, just tell me the one you saw most often)

Yes

No 🡪 question 60

Not sure 🡪 question 60

46. Briefly, why did you see this doctor? ________________________________ (Skip to 60 if not willing to allow access to medical records)

47. What was the name of your *doctor/midwife/etc*.?________________________

48. Did Dr. *name* work with a practice, group of doctors, or at a clinic?

Yes 🡪 What was the name of the *practice/clinic*? _________________

No, Dr. *name* worked alone

Not sure

49. Where was their office located?____________________(as specific as possible)

50. Do you happen to know the phone number for the *clinic*?__________________

51. How about an email or website address?________________________________

[repeat questions until no other providers mentioned]

[Skip to question 60]

**Miscarriage/abortion module**

52. Approximately how many weeks pregnant were you when you lost the pregnancy? ____

53. Did you go to the doctor or hospital for the miscarriage?

Yes

No

Not sure

We would like to know more about your health at the time of your miscarriage. For this reason, we are interested in looking at your medical records pertaining to this event. We are only interested in records pertaining to this event. If you give us permission to look at your records, we will mail a form to you for you to complete, sign, and return to us. This form gives your doctor permission to release these records to us. Your doctor will not release these records without your permission.

Willing or considering → question 54

Not willing → question 60

54. What was the name of your *doctor/midwife/etc.*? _________________________

55. Did *name* work with a practice or group of doctors?

Yes → What was the name of the *practice/clinic*? __________________

No

Not sure

56. Where was their office located? ______________________________________

57. Do you happen to know the phone number for the *clinic*? __________________

58. How about an email or website address? _______________________________

59. Approximately how many weeks pregnant were you when you ended the pregnancy? ____

**Concluding module**

60. Have you been pregnant any other time?

Yes

No 🡪 question 62

Not sure

61. When did you next get pregnant?

Age __________ or Year___________ 🡪 repeat questions 3-59 as appropriate

62. So, let me summarize, you’ve been pregnant ___ times and given birth to ___ children. Is that right? (If not, go back and correct.)

63. Have you had a period in the last year?

Yes

No

Not sure

64. Have you had your tubes tied (been sterilized)?

Yes

No

Not sure

65. Have you had your uterus removed (hysterectomy)?

Yes

No

Not sure

66. Have you ever been told by a doctor that you have polycystic ovarian syndrome (PCOS)?

Yes

No

Not sure

67. [Between the ages of 16 and 40], about how long was your average menstrual cycle (time from first day of one period to the first day of the next period)? Please do not include any time spent pregnant, receiving birth control pills or injections, after menopause, or after having both ovaries or the uterus surgically removed (select ONE only).

• <25 days

• 25-34 days

• 35-60 days

• More than 60 days

• Totally variable

68. [During the years you got your menstrual period](not including during pregnancy), did you have a tendency to grow dark, coarse hair on your:

• upper lip? Yes No

• chin? Yes No

• breasts? Yes No

• chest between the breasts? Yes No

• back? Yes No

• belly? Yes No

• upper arms? Yes No

• upper thighs? Yes No

69. [Between the ages of 16 and 40], did you ever notice a milky discharge from your nipples (not including during pregnancy or recent childbirth)?

Yes

No

Not sure

70. Did you ever try to get pregnant but were not able to?

Yes

No🡪 question 73

Not sure

71. How long were you trying? If it happened more than once, tell me the longest time. ____ months or ____ years

72. How old were you when this happened? ______

73. Have you ever been to a doctor to get help in getting pregnant?

Yes

No

Not sure

74. Have you ever had unprotected sex (that is, sex when you weren’t using birth control) for more than one year and not gotten pregnant?

Yes

No🡪 question 76

Not sure

75. How old were you when this happened? ______

76. How much do you weigh now?________

77. [*If no adult measure*] How much did you weigh when you were 20 years old? If you don’t remember, please tell me your best guess. _______

78. [*If no adult measure and >30 years*] How much did you weigh when you were 30 years old? If you don’t remember, please tell me your best guess. _______

**Family history module**

Now, I’m going to ask you a few questions about your family history.

79. How much did you weigh at birth?

__lb. __oz.

____g

Don’t know

80. Were you early, late, or on time when you were born?

Early

Late

On time

Don’t know 🡪question 82

81. How many days or weeks early or late were you? ____ days ____weeks

82. During any of her pregnancies, was your mother ever told by the doctor that she had diabetes (gestational diabetes)?

Yes

No

Not sure

83. During any of her pregnancies, was your mother ever told by the doctor that she had hypertension/high blood pressure (gestational hypertension)?

Yes

No

Not sure

84. During any of her pregnancies, was your mother ever told by the doctor that she had pre-eclampsia, eclampsia, or toxemia?

Yes

No

Not sure

85. Did your mother give birth to any babies that were less than 5 lbs?____

IF YES to 85: Were any of these small babies twins or triplets?

Yes, all of them

Yes, one/some of them

No

86. Did your mother give birth to any babies that were 3 weeks or more early?____

IF YES to 86: Were any of these early babies twins or triplets?

Yes, all of them

Yes, one/some of them

No

87. Did any of your mothers’ babies have to stay in the hospital for more than two days after the birth, or stay in the Neonatal Intensive Care Unit?

Yes

No

88. Do you have any sisters? For this question, we are only interested in sisters who have the same biological mother as you.

Yes

No 🡪 Go to question 96

89. Have any of your sisters ever been pregnant?

Yes

No 🡪next module

90. During any of their pregnancies, were any of your sisters ever told by the doctor that they had diabetes (gestational diabetes)?

Yes

No

Not sure

91. During any of their pregnancies, were any of your sisters ever told by the doctor that they had hypertension/high blood pressure (gestational hypertension)?

Yes

No

Not sure

92. During any of their pregnancies, were any of your sisters ever told by the doctor that they had pre-eclampsia, eclampsia, or toxemia?

Yes

No

Not sure

93. Did any of your sisters give birth to any babies that were less than 5 lbs?____

IF YES to 93: Were any of these small babies twins or triplets?

Yes, all of them

Yes, one/some of them

No

94. Did any of your sisters give birth to any babies that were 3 weeks or more early?____

IF YES to 94: Were any of these early babies twins or triplets?

Yes, all of them

Yes, one/some of them

No

95. Did any of your sisters’ babies have to stay in the hospital for more than two days after the birth, or stay in the Neonatal Intensive Care Unit?

Yes

No

[Ask to all]

96. How would you describe your family’s financial situation when you were a child?

Very poor, not enough to get by

Barely enough to get by

Have enough to get by but no extras

Have more than enough to get by

Well to do

97. How would you describe your family’s financial situation when you were a teenager?

Very poor, not enough to get by

Barely enough to get by

Have enough to get by but no extras

Have more than enough to get by

Well to do

98. How would you describe your family’s financial situation now?

Very poor, not enough to get by

Barely enough to get by

Have enough to get by but no extras

Have more than enough to get by

Well to do

99. What is the highest level of education your mother completed?

Less than elementary school

Elementary school

Junior high or middle school

Some high school

High school diploma

Associate’s degree

Some college

College degree

Some graduate school

Graduate degree

Don’t know

100. What is the highest level of education your father completed?

Less than elementary school

Elementary school

Junior high or middle school

Some high school

High school diploma

Associate’s degree

Some college

College degree

Some graduate school

Graduate degree

Don’t know

101. What is the highest level of education you have completed?

Less than elementary school

Elementary school

Junior high or middle school

Some high school

High school diploma

Associate’s degree

Some college

College degree

Some graduate school

Graduate degree

102. What is your date of birth? ___/___/_____

103. Please provide us with any other names you have gone by in the past. ____________________________________________________________

104. What is your race?

White

Black

Asian

Other: ___________

105. Do you consider yourself Hispanic or Latina?

Yes

No
